# Individualized Risk Prediction Model for Lung Cancer in Korean Men

**DOI:** 10.1371/journal.pone.0054823

**Published:** 2013-02-07

**Authors:** Sohee Park, Byung-Ho Nam, Hye-Ryung Yang, Ji An Lee, Hyunsun Lim, Jun Tae Han, Il Su Park, Hai-Rim Shin, Jin Soo Lee

**Affiliations:** 1 Biometric Research Branch, National Cancer Center, Goyang, Republic of Korea; 2 Department of Epidemiology and Health Promotion, Graduate School of Public Health, Yonsei University, Seoul, Republic of Korea; 3 Gangnam Biomedical Research Center, Yonsei University College of Medicine, Seoul, Republic of Korea; 4 Information Team, Ministry of Patriots & Veterans Affairs, Seoul, Republic of Korea; 5 Department of Health, Uiduk University, Gyeongju, Republic of Korea; 6 Division of Cancer Registration and Surveillance, National Cancer Center, Goyang, Republic of Korea; 7 Western Pacific Regional Office, World Health Organization, Manila, Philippines; 8 Center for Lung Cancer, National Cancer Center, Goyang, Republic of Korea; The University of Texas M. D. Anderson Cancer Center, United States of America

## Abstract

**Purpose:**

Lung cancer is the leading cause of cancer deaths in Korea. The objective of the present study was to develop an individualized risk prediction model for lung cancer in Korean men using population-based cohort data.

**Methods:**

From a population-based cohort study of 1,324,804 Korean men free of cancer at baseline, the individualized absolute risk of developing lung cancer was estimated using the Cox proportional hazards model. We checked the validity of the model using C statistics and the Hosmer–Lemeshow chi-square test on an external validation dataset.

**Results:**

The risk prediction model for lung cancer in Korean men included smoking exposure, age at smoking initiation, body mass index, physical activity, and fasting glucose levels. The model showed excellent performance (C statistic = 0.871, 95% CI = 0.867–0.876). Smoking was significantly associated with the risk of lung cancer in Korean men, with a four-fold increased risk in current smokers consuming more than one pack a day relative to non-smokers. Age at smoking initiation was also a significant predictor for developing lung cancer; a younger age at initiation was associated with a higher risk of developing lung cancer.

**Conclusion:**

This is the first study to provide an individualized risk prediction model for lung cancer in an Asian population with very good model performance. In addition to current smoking status, earlier exposure to smoking was a very important factor for developing lung cancer. Since most of the risk factors are modifiable, this model can be used to identify those who are at a higher risk and who can subsequently modify their lifestyle choices to lower their risk of lung cancer.

## Introduction

Lung cancer is one of the most commonly occurring malignancies, with more than 1.3 million incident cases, and a major cause of cancer death worldwide [1]. In Korea, cancer has been the leading cause of death since the 1980s; in particular, lung cancer has ranked first among all cancer deaths. In 2010, a total of 15,623 lung cancer deaths occurred, and 73% (n = 11,411) of them were among men in Korea [2]. Lung cancer was also the second most common cancer in incidence among Korean men and the first among elderly Korean men (≥65 years). The age-standardized lung cancer incidence rates in 2009 were 46.8 in men and 13.9 in women per 100,000 person-years [3]. Compared with other types of cancer, lung cancer survival rates were much lower in Korea. The 5-year relative survival rates were 14.9% in men and 19.6% in women for cancer patients newly diagnosed between 2001 and 2005 in Korea [3].

The established risk factors for lung cancer include active tobacco smoke, second-hand smoke, air pollution, industrial chemicals, physical inactivity, and a family history of lung cancer [4]–[6]. The consumption of fruits and vegetables, especially those containing beta-carotene or carotenoids, has been shown to reduce the lung cancer risk [7]. On the other hand, an increased risk of lung cancer was reported in the Carotene and Retinol Efficacy Trial (CARET), particularly in high-risk populations including heavy smokers [8]. Among these risk factors, smoking is known to be the most important factor that can be modified at the individual level. Historically smoking prevalence has been high among Korean men. Although it has continuously decreased over the last two decades, from 75.1% in 1992 to 43.1% in 2009, the smoking prevalence in Korean men is still among the highest of countries included in the Organisation for Economic Cooperation and Development (OECD) [9], [10]. Smoking patterns and the magnitude of the increased risk of lung cancer among smokers in Asian populations are very different from those in Western populations. The lung cancer risks observed among smokers in Asia are generally much lower than those among smokers in Western populations. The relative risk for current smokers in Western countries reportedly ranges from 9.94 [11] to 12.8 [12], whereas in Asia it is as low as 4.0 [13].

To identify those with a higher risk of lung cancer for the purposes of prevention and early detection, the development of an individualized risk prediction model for lung cancer is vital. Several lung cancer risk prediction models have been developed, however they are predominantly focused on participants in Western populations from the United States [14]–[19]. No previous study has developed an absolute risk prediction model for lung cancer that can be directly applied to an Asian population. Therefore, there is a great need to develop and validate a population-specific risk prediction model using data from Asian countries. The objective of the present study was to develop a lung cancer risk prediction model in Korean men using a large population-based prospective study.

## Methods

### Ethics Statement

This study was approved by the Institutional Review Board of the National Cancer Center, Korea (IRB no. NCCNCS09-305). The need for participants’ consent was waived by the ethics committee because this study involved routinely collected medical data that were anonymously managed in all stages, including data cleaning and statistical analyses.

### Study Population and Data Collection

All men between the ages of 30 and 80 years who underwent health examinations between 1996 and 1997 were used for this study. During the health check-ups, participants filled out a questionnaire about smoking habits, alcohol drinking, physical activity, meal preferences (meat vs. vegetables), previous disease history, and history of disease in parents or siblings (including any type of cancer, cardiovascular disease, or diabetes). Height, weight, and blood pressure were directly measured. Smoking status was classified as never, past, and current smoker. A past smoker was defined as a person who “has quit smoking for at least 1 year” before the time of the health check-up. Duration of smoking was assessed for past and current smokers, and the average amount smoked per day was assessed for current smokers. Physical activity was evaluated based on the intensity (number of exercise sessions per week) and duration (how long per session) of leisure-time physical activity. We classified physical activity into three groups: (1) low, ≤4 times/week at <30 minutes/session; (2) moderate, 2–4 times/week at ≥30 minutes/session or ≥5 times/week at <30 minutes/session; and (3) high, ≥5 times/week at ≥30 minutes/session [20], [21].

Blood and urine laboratory test results were obtained, including fasting glucose levels. The health check-up data were obtained from the Korea National Health Insurance Corporation.

Cancer incidence cases among study participants were identified through the Korea Central Cancer Registry database. The Korea Central Cancer Registry is a combination of a hospital- and population-based cancer registry system covering more than 95% of all newly diagnosed cancer cases in Korea. Lung cancer cases were classified according to ICD-10 codes (C33 and C34) [22]. Information on participants’ vital status was obtained from the death certification data of the Korean Statistics Office [2]. The starting point was the date of health examination, the event date was the date of first diagnosis of lung cancer, and the last date of follow-up was December 2007. Participants free of lung cancer until the end of follow-up were considered censored. We restricted our analyses to participants aged 30 to 80 years who were free of any cancer at baseline and for whom we had complete information on the relevant risk factors.

### Development of the Risk Prediction Model

Our model was configured to estimate the absolute risk that an individual will have lung cancer in 8 years. To identify the significant risk factors for lung cancer in our data, we explored the crude and age-adjusted analysis for each risk factor. We employed the Cox proportional hazards model to develop a multivariable model for lung cancer risk. The time to event was defined as the difference between the date of health examination at baseline and the date of first lung cancer diagnosis or follow-up termination, whichever came first. To select the best-fit risk prediction model for lung cancer, we included variables that showed statistical significance at the 0.10 level in the univariate analysis or that were chosen from the stepwise regression model. We then used a hierarchical variable selection method by comparing models with different sets of variables. Likelihood ratio tests were employed to select significant variables. The proportional hazards assumption was checked by investigating the log-log survival plot. Age was included in the model as a quadratic term (age-mean_age_ and [age-mean_age_]^2^) because it improved the model fit. All other variables were included in the model as categorical variables. We also considered a composite variable for smoking that combined smoking status and the average amount smoked per day (non-smoker, past smoker, or current smoker consuming <0.5 pack/day; current smoker consuming 0.55–0.99 packs/day; or current smoker consuming >1 pack/day). For body mass index (BMI), we used the World Health Organization (WHO) criteria specific to Asian populations (<18.5, 18.5–22.9, 23.0–24.9, and ≥25.0) [23]. Age at smoking initiation was assessed using the information on smoking duration for current smokers only and age at the time of the questionnaire. We divided the age at smoking initiation into five age groups (<16, 16–18, 19–29, 30–39, and ≥40 years) based on the school aging system in Korea and 10-year age intervals. For the category of “<16 years age at smoking initiation,” we first considered a finer division such as <11, 12–13, and 14–15 years; however, due to lack of sufficient cases in these age groups, we used the collapsed category.

A simple prediction model including only age and smoking variables was also considered. Both age and smoking variables were highly significant predictive variables, and the estimated hazard ratios were very similar to those in the multivariable model with additional variables. However, the likelihood ratio test revealed that the model with additional variables had an improved model fit compared with a simple model including age and smoking only (likelihood ratio test, χ^2^ = 442.14, df = 11, p<0.0001). Our final model for predicting individualized lung cancer risk included age, smoking status, age at smoking initiation, BMI, physical activity, and fasting glucose levels.

The probability of developing lung cancer within 8 years (t = 8) was estimated as follows:
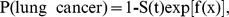
where f(x) = β_1_x_1_+ β_2_x_2_+ β_3_x_3_+…+β_k_x_k_.

In the above equation, x_1_,…, x_k_ are the values of risk factors, M_1_,…, M_k_ are the mean values for relevant risk factors, and β_1_,…, β_k_ are the coefficient estimates from the Cox proportional hazard model. Baseline survival probability at time t (t = 8 years), S(t), is estimated when all risk factors are at their mean values. Based on the β coefficients from the Cox proportional hazard model, score sheets were developed by assigning points for each risk factor [24]. The detailed scoring system for our lung cancer risk prediction model is presented in Appendix S1.

### Validation of the Risk Prediction Model

We tested the validity of our model with an external validation using participants from the Korean National Health Corporation (1998 to 1999). Data of 507,046 male participants were used in the validation analysis. Performance of the model was evaluated with respect to discrimination and calibration. Discrimination was quantified using the C-statistics developed for survival data [25]. C-statistic is a concordance measure, analogous to the Receiver Operating Characteristic (ROC) curve area which can be interpreted as the probability that the model predicts a higher risk of lung cancer for those who actually developed lung cancer compared with those who did not develop lung cancer over the follow-up time [26]. The prediction model is considered good when the discrimination is >0.75. Calibration ability refers to how closely the predicted probabilities agree numerically with the actual outcomes. We used a Hosmer–Lemeshow (H-L)-type χ^2^ statistic developed for survival data [25]. The risk of developing lung cancer for each participant was calculated from the prediction model, and results were sorted in ascending order. Then, in each decile, the average predicted probabilities were compared with the actual lung cancer risk estimated by the Kaplan–Meier approach. The performance of the developed model was also tested on the external validation dataset in regard to discrimination and calibration. Statistical analyses were performed using SAS version 9.1 (SAS institute, Cary, NC), and graphs were generated using STATA statistical software, Version 10 (STATA, College Station, TX).

## Results

### Major Risk Factors Affecting the Lung Cancer Risk

A total of 1,309,144 participants aged 30 to 80 years were included in this study, and 10,007 newly diagnosed lung cancer cases were observed during the 8-year follow-up. Table 1 presents the number of newly diagnosed lung cancer cases in this study and compares the lung cancer rates in our cohort with those in the total Korean male population. While slightly higher than those in the general population in Korea, the age-specific lung cancer incidence rates in this study appeared to be representative of the Korean male population (Table 1).

**Table 1:** Comparison of lung cancer incidence rates in our study and in the total Korean male population.

	Cohort in this study				Total Korean male population
Age	Total number	Person-years (pyrs)	Newly diagnosed lung cancer cases	Lung cancer incidence rate (/100,000 pyrs)†	Lung cancer incidence rate† (/100,000 pyrs)
30–34	236,521	2,674,905	111	4.2	1.5
35–39	251,249	2,834,072	257	9.2	3.5
40–44	245,257	2,729,318	598	21.9	9.9
45–49	179,481	1,984,644	1,033	52.2	20.3
50–54	149,536	1,628,114	1,537	94.4	44.9
55–59	120,381	1,277,575	2,104	164.7	104.1
60–64	72,428	731,329	2,090	285.8	197.4
65–69	29,232	270,031	1,170	433.3	350.7
70–74	17,051	142,821	793	555.2	513.4
75–80	8,008	58,725	314	534.7	655.7
Total	1,309,144	14,331,533	10,007	69.83	-

† Ministry of Health, Welfare and Family Affairs. Annual report of cancer incidence (2005) and survival (1993–2005) in Korea, 2008; Cancer incidence in 2005 was used for comparison with study participants with follow-up period of 1996–2007.

The mean age of the cohort was 45 years. A total of 28.6% were never smokers, and 13.9% were current smokers consuming ≥1 pack per day. The majority of participants were alcohol consumers (84.6%) and had a BMI within the normal range (18.5–24.9, 69.0%). Twelve percent of participants had a family (parent or sibling) history of any cancer, and 6% had fasting glucose levels >126 mg/dL (Table 2).

**Table 2:** Baseline characteristics and univariate analysis.

Risk factor		Total (*N* = 1,309,144)	Events (*n* = 10,007)	Hazard ratio* (95% CI)
Age, mean (SD), y		44.66 (10.36)		
Age	≤50 years	942,632 (72.00%)	2,231 (22.29%)	1.00 (reference)
	>50 years	366,512 (28.00%)	7,776 (77.71%)	1.66 (1.54–1.78)
Smoke	Never	374,917 (28.64%)	1,535 (15.34%)	1.00 (reference)
	Past	197,813 (15.11%)	1,287 (12.86%)	1.52 (1.41–1.63)
	Current, <0.5 pack/day	118,504 (9.05%)	896 (8.95%)	1.87 (1.73–2.04)
	Current, 0.5–0.99 pack/day	435,914 (33.30%)	3,956 (39.53%)	3.34 (3.15–3.55)
	Current, ≥1 pack/day	181,996 (13.90%)	2,333 (23.31%)	5.33 (5.00–5.69)
Age at smoking initiation	Never or Past	572,730 (43.75%)	2,822 (28.20%)	0.54 (0.51–0.58)
	Age ≥40	50,567 (3.86%)	1,283 (12.82%)	1.00 (reference)
	30≤ Age <40	188,622 (14.41%)	2,995 (29.93%)	1.76 (1.64–1.88)
	19≤ Age <30	467,375 (35.70%)	2,740 (27.38%)	1.91 (1.77–2.07)
	16≤ Age <19	21,495 (1.64%)	130 (1.30%)	2.38 (1.98–2.88)
	Age <16	8,355 (0.64%)	37 (0.37%)	2.54 (1.82–3.54)
Alcohol	Non-drinker (0 g/week)	201,626 (15.40%)	1,353 (13.52%)	1.00 (reference)
	Low (1–188.9 g/week)	605,688 (46.27%)	3,692 (36.89%)	1.09 (1.03–1.17)
	Medium (189–440.9 g/week)	195,467 (14.93%)	1,512 (15.11%)	1.30 (1.21–1.40)
	High (≥441 g/week)	117,112 (8.95%)	1,086 (10.85%)	1.58 (1.45–1.71)
BMI, kg/m^2^	<18.5	31,252 (2.39%)	627 (6.27%)	1.41 (1.30–1.54)
	18.5–22.9	533,254 (40.73%)	4,928 (49.25%)	1.00 (reference)
	23–24.9	371,454 (28.37%)	2,401 (23.99%)	0.74 (0.70–0.77)
	≥25.0	373,184 (28.51%)	2,051 (20.50%)	0.65 (0.62–0.69)
Physical activity	None	626,088 (47.82%)	5,449 (54.45%)	1.000 (reference)
	Light	208,146 (15.90%)	1,358 (13.57%)	0.86 (0.81–0.91)
	Moderate	386,403 (29.52%)	2,269 (22.67%)	0.78 (0.74–0.82)
	Heavy	88,507 (6.76%)	931 (9.30%)	0.89 (0.83–0.95)
Family history of cancer	No	676,870 (51.70%)	5,642 (56.38%)	1.00 (reference)
	Yes	152,786 (11.67%)	1,002 (10.01%)	0.98 (0.92–1.05)
Fasting glucose level, mg/dL	<126	1,229,881 (93.95%)	9,049 (90.43%)	1.00 (reference)
	≥126	79,263 (6.05%)	958 (9.57%)	1.10 (1.03–1.18)

* Hazard ratios were obtained from a Cox proportional hazards model.

In univariate analyses, older age, smoking, early age at smoking initiation, high alcohol consumption, and low BMI were significantly associated with a higher lung cancer risk. Having a family history of any cancer was not significantly related to lung cancer risk. High glucose levels were also associated with an elevated lung cancer risk. In the multivariable setting, age was included as a quadratic term (age2) in the model. Smoking duration did not improve the goodness of fit of the model; thus, in the final model, we used a composite of variables for smoking, which was divided into five categories (never; past; current, <0.5 packs/day; current, 0.5–0.99 packs/day; and current, ≥1 pack/day) based on the combination of smoking status and the average amount smoked per day. Current smokers with high cigarette consumption (≥1 pack/day) showed an approximately four-fold elevated risk of developing lung cancer, and there was a significant increasing trend of lung cancer risk by amount smoked (*p* for trend <0.0001). Alcohol consumption was no longer significant when it was included in the model simultaneously with smoking; hence, it was excluded from our final model.

Lean participants (BMI <18.5) had a 39% increased risk of developing lung cancer, whereas heavier participants had an approximately 29% decreased risk compared with participants with a normal BMI. Physical activity appeared to decrease the lung cancer risk by about 5–13%, and high fasting glucose levels (≥126 mg/dL) was significantly associated with lung cancer (Table 3).

**Table 3:** Multivariable regression model: risk prediction model.

Risk factor	β	HR* (95% CI)	*p*-value	*p* for trend
Age-Mean_age_, years	0.1668	1.18 (1.18–1.19)	<0.0001	
(Age-Mean_age_)^2^, years^2^	−0.0020	1. 00 (1.00–1.00)	<0.0001	
Smoke				
Never		1.00 (reference)		<0.0001
Past	0.4180	1.52 (1.41–1.64)	<0.0001	
Current, <0.5 pack/day	0.4444	1.56 (1.42–1.71)	<0.0001	
Current, 0.5–0.99 pack/day	0.9414	2.56 (2.37–2.78)	<0.0001	
Current, ≥1 pack/day	1.3889	4.01 (3.68–4.37)	<0.0001	
Age at smoking initiation				
Age ≥40		1.000 (reference)		<0.0001
30≤ Age <40	0.2194	1.25 (1.16–1.34)	<0.0001	
19≤ Age <30	0.2809	1.32 (1.23–1.43)	<0.0001	
16≤ Age <19	0.5249	1.69 (1.4–2.04)	<0.0001	
Age <16	0.7120	2.04 (1.46–2.84)	<0.0001	
BMI, kg/m^2^				
<18.5	0.3306	1.39 (1.28–1.51)	<0.0001	<0.0001
18.5–22.9		1.00 (reference)		
23.0–24.9	−0.2468	0.78 (0.74–0.82)	<0.0001	
≥25.0	−0.3386	0.71 (0.68–0.75)	<0.0001	
Physical activity				
No		1.000 (reference)		<0.0001
Light	−0.0909	0.91 (0.86–0.97)	0.0029	
Moderate	−0.1412	0.87 (0.83–0.91)	<0.0001	
Heavy	−0.0521	0.95 (0.89–1.02)	0.1431	
Fasting glucose level, mg/dL				
<126		1.000 (reference)		
≥126	0.0792	1.08 (1.01–1.16)	0.0201	0.0201

* Hazard ratios were obtained from a Cox proportional hazards model.

### Age at Smoking Initiation

**Figure 1:**
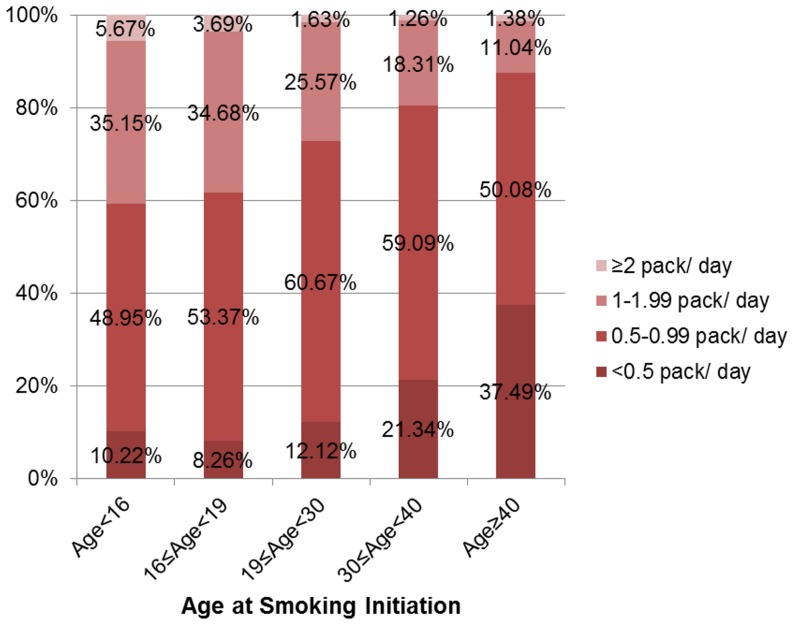
Association between age at smoking initiation and the smoking amount per day among current smokers.

Our data also showed that the age at smoking initiation was significantly associated with lung cancer risk (Tables 2 and 3). The younger the age of smoking initiation the higher the risk of lung cancer. Furthermore, the age at smoking initiation was shown to be negatively associated with the average amount smoked per day (Figure 1). Age at smoking initiation remained significant in the multivariable regression model and was therefore selected for the final model.

### Score Sheet for Lung Cancer Risk

The predicted probability of developing lung cancer in 8 years was calculated based on the scoring system presented in Appendix S1 A. The score sheets reflected the standardized point-based score system for each risk factor in the final model (Appendix S1 B). The standardized points for each risk factor were calculated to be proportional to the β coefficients from the risk prediction model and rounded up to the nearest integer.

### Validation of the Risk Prediction Model

**Figure 2:**
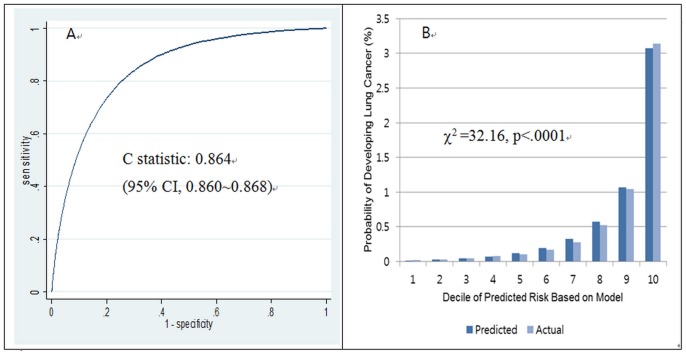
Discrimination and calibration of the lung cancer prediction model (A: discrimination, B: calibration).

Our risk prediction model showed excellent discrimination (C statistic = 0. 864, 95% CI = 0.860–0.868) (Figure 2). The prediction model with only age and smoking variables also showed excellent discrimination (C statistic = 0.861, 95% CI = 0.857–0.865). However, the model fit was improved by including other covariates (age at smoking initiation, physical activity, BMI, and fasting glucose levels), therefore our final model included all significant variables (likelihood ratio test, χ^2^ = 442.14, df = 11, p<0.0001). While the discrimination of the model was excellent, the calibration was rather limited (Hosmer–Lemeshow type χ^2^ test, p<0.001), as shown in Figure 2.

**Figure 3:**
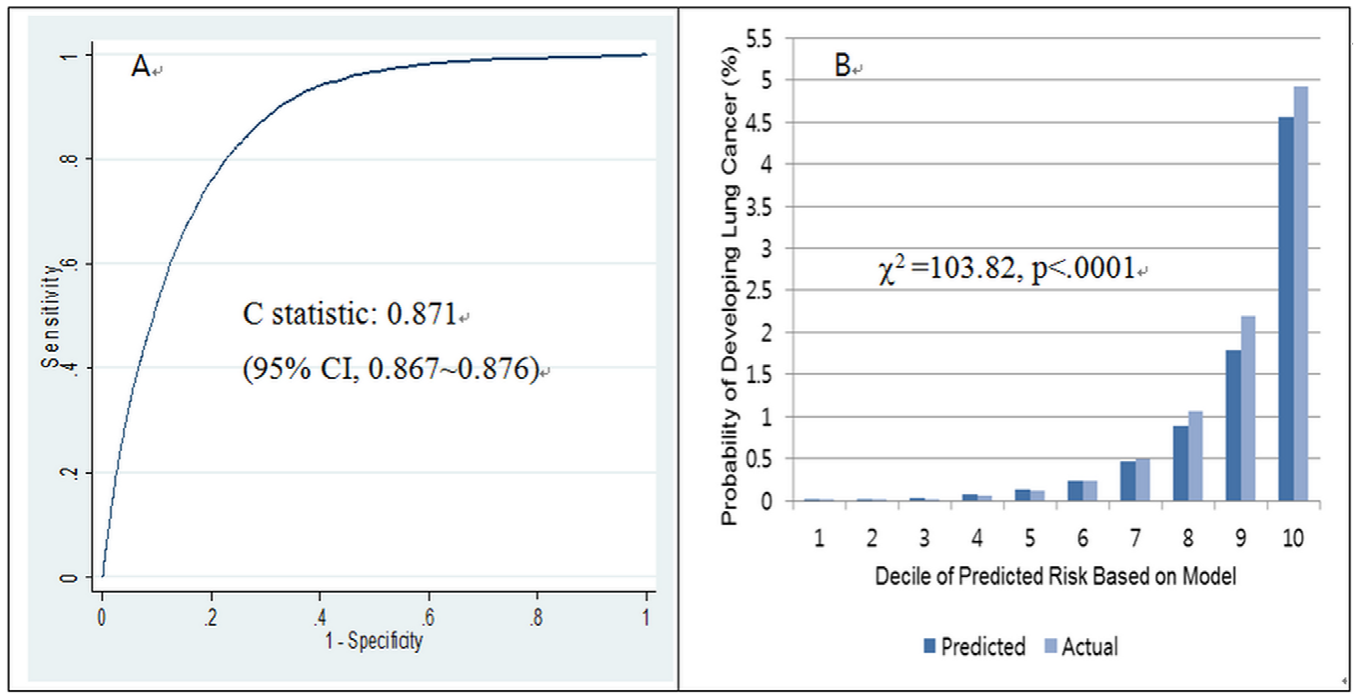
Discrimination and calibration of the lung cancer prediction model tested on an external validation set* (A: discrimination, B: calibration, *n* = 507,046, event = 4,539).

When the performance of our developed model was tested on an external validation dataset, the discrimination was excellent (C statistic = 0.871, 95% CI = 0.867–0.876), as shown in Figure 3.

### Illustration of Individual Absolute Risk Estimate for Lung Cancer

Table 4 presents the estimated probability of developing lung cancer within 8 years in Korean men with various age and risk profiles. The first risk profile is for a man with the lowest combination of risk factors. He is a 30-year-old man who has never smoked in his lifetime, has a BMI of ≥25 with moderate physical activity, and has fasting glucose levels in the normal range. The absolute risk of developing lung cancer within 8 years for this person is only 0.004%. In contrast, for risk profile #9, a 65-year-old man who is a current smoker consuming ≥1 pack of cigarettes per day, started smoking at <16 years of age, is thin (BMI of <18.5), has low physical activity, and has glucose levels above the normal range (≥126 mg/dL), the absolute risk of getting lung cancer in 8 years is as high as 22.31%. This is about 16 times (22.31% vs. 1.39%) higher than the risk for an identically aged man with the lowest risk who has a “preventive” lifestyle such as never smoking, doing moderate physical activity, and maintaining normal body weight and health conditions such as normal fasting glucose levels (risk profile #12).

**Table 4:** Illustration of 8-year absolute risk estimates for lung cancer in Korean men with different risk factor profiles.

Patient profile no.	Age (years)	Smoking	Age at smoking initiation	BMI	Physical activity	Fasting glucose level (mg/dL)	8-year absolute risk (%)
1	30	Current, ≥1 pack	Age <16	<18.5	No	≥126	0.081
2	30	Current,<0.5 pack	16≤ Age <19	18.5–22.9	Light	<126	0.0160
3	30	Past	–	≥25.0	Moderate	<126	0.006
4	30	Non-smoker	–	≥25.0	Moderate	<126	0.004
5	50	Current, ≥1 pack	Age <16	<18.5	No	≥126	3.228
6	50	Current,<0.5 pack	16≤ Age <19	18.5–22.9	Light	<126	0.639
7	50	Past	–	≥25.0	Moderate	<126	0.250
8	50	Non-smoker	–	≥25.0	Moderate	<126	0.165
9	70	Current, ≥1 pack	Age <16	<18.5	No	≥126	24.311
10	70	Current,<0.5 pack	16≤ Age <19	18.5–22.9	Light	<126	5.298
11	70	Past	–	≥25.0	Moderate	<126	2.104
12	70	Non-smoker	–	≥25.0	Moderate	<126	1.390

### Smoking Cessation Effect

Table 5 illustrates the range of predicted 8-year lung cancer risk among smokers in various percentiles according to the modification of smoking status. For a 57-year-old Korean man who smokes more than half a pack of cigarettes per day, started smoking in his thirties, is overweight, has low physical activity, and has normal blood glucose levels, the lung cancer risk is in the 95th percentile. If he quits smoking, his risk of developing lung cancer within the next 8 years is 0.73% as opposed to 2.51% if he continues to smoke, which is about 3.47 times higher. A similarly large discrepancy in lung cancer risk was estimated between men that quit smoking and those who have not. For instance, for a 33-year-old man who currently smokes ≥1 pack of cigarettes per day, began smoking between 19 and 30 years of age, is overweight, performs light physical activity, and has normal fasting glucose levels, the present risk of lung cancer is in the 25th percentile among all participants. If he quits smoking, his risk of developing lung cancer within the next 8 years is 0.013% as opposed to 0.04% if he continues to smoke, which is about 3.50 times higher (Table 2).

**Table 5:** Predicted lung cancer risk within 8 years by modification of smoking habits among smokers.

	Percentile of lung cancer risk				
Risk factor	5th	25th	50th	75th	95th
Age (years)	34	33	42	46	57
Smoking	Never	≥1 pack/day	0.5–0.99 pack/day	≥1 pack/day	≥1 pack/day
Age at smoking initiation	NA	19≤ Age <30	30≤ Age <40	30≤ Age <40	19≤ Age <30
BMI	23.0–24.9	≥25.0	≥25.0	23.0–24.9	≥25.0
Physical activity	No	Light	Light	No	No
Glucose, mg/dL	<126	<126	<126	<126	<126
8-year risk if no further smoking, % (A)	NA	0.013	0.072	0.171	0.726
8-year risk if continued smoking, % (B)	NA	0.044	0.152	0.561	2.514
Ratio (B/A)	NA	3.496	2.101	3.282	3.465

† NA = not applicable.

## Discussion

In this study, we developed a lung cancer risk prediction model using the data from a large-scale population-based cohort of Korean men and examined the model performance using an external validation dataset. To our knowledge, this is the first comprehensive effort to develop an absolute lung cancer risk prediction model that also evaluated the discrimination and calibration of the model with an external validation data in the Asian population. Our model discriminates well between patients with lung cancer and normal controls with a C-statistic of 0.864. Many lung cancer risk prediction models have been previously developed. Spitz et al. utilized participants from a case-control study at The University of Texas MD Anderson Cancer Center [17]; Colditz et al. used Surveillance Epidemiology and End Results (SEER)data [27], Bach et al. used CARET data [28], and and Tammenagi et al, recently published their findings using participants from the Prostate, Lung, Colorectal, and Ovarian Cancer Screening Trial (PLCO) [18]. However, all these studies were conducted with White participants in the United States. Spitz et al further recognized the importance of race-specific risk prediction models and developed an African American risk prediction model for lung cancer. [16]. In regard to discrimination, our model showed a higher discrimination (86%) than did most other lung cancer risk models, which have shown discrimination ranging from 57% [17] to 75% [16] and discrimination similar to that in the PLCO trial (86%) [18].

Smoking patterns and the magnitude of increased risk of lung cancer among smokers are very different between Asian and Western populations. The lung cancer risks observed among smokers in Asian populations are in general much lower than those in Western populations. Meta-analysis results by Gandini et al. showed that White and African-American smokers are at 9.94- and 10.2-fold higher risk of having lung cancer compared with non-smokers, respectively [11]. In contrast, the lung cancer risk among current smokers is about four times the risk among non-smokers in Asian countries such as Japan, China, and Korea [13]. Furthermore, lung cancer incidence rates in American men have greatly exceeded those in Japanese men for several decades despite the higher smoking prevalence in Japanese men, also known as the “Japanese smoking paradox” [29]. A multicenter case–control study involving both American and Japanese individuals was carried out and showed striking results: the odds ratio (OR) of current US smokers relative to non-smokers was 40.4, which is more than 6 to 10 times higher than the OR for current Japanese smokers (3.5–6.3) [30]. Several possible explanations for the differences in OR between Asian and Western populations have been suggested, including a longer duration of heavy smoking in Americans, a more toxic formulation of American-manufactured cigarettes, higher efficiency of filters in Japanese cigarettes, lower alcohol consumption by Japanese males, and a higher background risk of lung cancer among non-smokers in Asia [31]. The lung cancer mortality rates per 100,000 person-years among non-smokers in Asian populations (rate = 35.6 in Japanese men and 24.6 in Japanese women) were indeed shown to be much higher than those in the US (rate = 15.7 in Cancer Prevention Study I (CPS-I) and 14.7 in Cancer Prevention Study II (CPS-II)) [12], [21], [32]. In our study, the crude lung cancer incidence rate among non-smokers was 37.28 per 100,000 person-years, which appears to be very similar to rates in Japan and higher than rates in Western countries. A possible explanation for these higher lung cancer incidence rates among non-smokers in Asian countries is that they are more exposed to indoor air pollution and secondhand smoke, and the background risk of lung cancer is elevated among Asian non-smokers [33].

Since Korea has universal health insurance coverage by the Korea National Health Insurance Corporation (KNHIC), the algorithm of this individualized prediction model for lung cancer can be utilized in the KNHIC database and the results can be provided to health examinees when they receive their health check-up results. This will be helpful when clinicians counsel patients and recommend lifestyle modifications, most importantly to quit smoking (or to continue not smoking or not to begin smoking). Age at smoking initiation was shown to be negatively associated with the average amount smoked per day. This might be attributable to the fact that participants who began smoking at an earlier age tend to be more addicted to nicotine, hence the average number of cigarettes consumed per day is higher in these participants. In our model the lung cancer risk for these participants was higher.

Another interesting feature of our model is its inclusion of BMI. Our data showed a very consistent inverse relationship between level of body fat (BMI) and lung cancer risk. Because we anticipated potential residual confounding by smoking, we also performed subset analyses on BMI separately in non-smokers, past smokers, and current smokers. However, the inverse relationship still remained significantly strong (p for trend, <0.001). To avoid the possible bias derived from participants who had lower weights at baseline due to existing progressive lung cancer, we repeated our analyses excluding all lung cancer cases diagnosed within the first 1 or 2 years after initiation of the study. In this sensitivity analysis, the same trend with respect to BMI was observed. According to a systematic review of 21 cohort studies, 24 case–control studies, and 1 ecological study that investigated body fat and lung cancer risk, 20 cohort studies showed a decreased lung cancer risk with increased BMI, 12 of which showed statistically significant results. The meta-analysis suggested a 5% decreased risk of lung cancer for each increase of 5 kg/m2 [7]. Our analysis showed consistent results in all three groups divided by smoking status; hence, this effect of BMI was too strong to ignore or to regard as an artifact derived from confounding with smoking. The possibility of weight loss in patients with undiagnosed cancer remains questionable. The effect of physical activity appeared to be stronger among non-smokers in our subset analysis.

The performance of the models was measured with respect to discrimination and calibration abilities. Furthermore, unlike in the risk prediction model developed from a case–control study and baseline incidence rates, our study was based on a population-based cohort; hence, modification of the model, such as updating the risk factors and including newly diagnosed cancer cases, was possible. We also validated our model with an external dataset.

There are several potential limitations of this study. First, there was no assessment of the effect of environmental or occupational risk factors on lung cancer, such as second-hand smoke, exposure to air pollution or asbestos, etc. However, because lung cancer risk is mostly dominated by active cigarette smoking (C-statistic = 0.861 when the model includes age and smoking variables only) and this model was developed for Korean men, whose adult smoking prevalence is high, we believe that the impact of not including environmental risk factors in our model is minimal. Second, the information on family history of lung cancer was not available in the data used to develop the risk prediction model in this study. Third, this lung cancer risk prediction model included only men. The smoking prevalence among Asian women is very different from that among Asian men, usually much lower. In Korea, smoking among women is not culturally well accepted; thus, the reporting of smoking habits is known to be underestimated. However, even taking into account the underreporting of smoking among women, the cancer burden attributable to men is much greater in Korea. We believe that risk prediction models should be developed in men and women separately. A model for women was not developed in this study because there was insufficient smoking data for women. Finally, although smoking status and smoking intensity among current smokers were considered in the model, we were not able to differentiate the effect of smoking intensity among past smokers because such data were not available.

Despite the limitations mentioned above, we believe that our study provides a very important tool, namely, the first Asian version of a validated lung cancer risk prediction model that can project the absolute risk of developing lung cancer. Projection of an individual’s absolute risk can be estimated only by prediction equations developed from a longitudinal study, and in this sense, our risk prediction model is of great importance. It is expected to play an important role in applying cancer prevention strategies in Korea and can provide a further reference for other Asian populations.

## Supporting Information

Appendix S1 **Detailed scoring system for our lung cancer risk prediction model.** Table A. Application of Tables. Table B. Score sheets were developed to predict the lung cancer risk from the β-coefficient estimates in the Cox regression model (Table 3).
(DOCX)

## References

[1] Parkin DM, Bray F, Ferlay J, Pisani P (2005) Global cancer statistics, 2002. CA Cancer J Clin 55: 74–108. 15761078 10.3322/canjclin.55.2.74

[2] Statistics Korea (2010) Causes of deaths in Korea, 2010. Statistics Korea.

[3] Jung KW, Park S, Kong HJ, Won YJ, Lee JY, et al (2012) Cancer statistics in Korea: incidence, mortality, survival, and prevalence in 2009. Cancer Res Treat 44: 11–24. 22500156 10.4143/crt.2012.44.1.11 PMC3322196

[4] Secretan B, Straif K, Baan R, Grosse Y, El Ghissassi F, et al (2009) A review of human carcinogens Part E: tobacco, areca nut, alcohol, coal smoke, and salted fish. Lancet Oncol 10: 1033–1034. 19891056 10.1016/s1470-2045(09)70326-2

[5] Doll R, Peto R (1981) The causes of cancer: quantitative estimates of avoidable risks of cancer in the United States today. J Natl Cancer Inst 66: 1191–1308. 7017215

[6] Schottenfeld D, Fraumeni JF (2006) Cancer Epidemiology and Prevention: Oxford University Press.

[7] Ells L, Summerbell C, Kelly S, Hillier F, Smith S, et al.. (2007) Food, nutrition, physical activity, and the prevention of cancer: a global perspective.

[8] Omenn GS (2007) Chemoprevention of lung cancers: lessons from CARET, the beta-carotene and retinol efficacy trial, and prospects for the future. Eur J Cancer Prev 16: 184–191. 17415088 10.1097/01.cej.0000215612.98132.18

[9] Ministry of Health, Welfare and Family Affairs, editor (2009) National survey on smoking prevalence and behavior.

[10] American Cancer Society, editor (2009) The Tobacco Atlas.

[11] Gandini S, Botteri E, Iodice S, Boniol M, Lowenfels AB, et al (2008) Tobacco smoking and cancer: a meta-analysis. Int J Cancer 122: 155–164. 17893872 10.1002/ijc.23033

[12] Thun M, Day-Lally C, Myers D, Calle E, Flanders W, et al. (1996) Trends in tobacco smoking and mortality from cigarette use in Cancer Prevention Studies I (1959 through 1965) and II (1982 through 1988): Smoking and Tobacco Control Monograph 8.

[13] Jee SH, Yun JE, Park EJ, Cho ER, Park IS, et al (2008) Body mass index and cancer risk in Korean men and women. Int J Cancer 123: 1892–1896. 18651571 10.1002/ijc.23719

[14] Bach P, Kattan M, Thornquist M, Kris M, Tate R, et al (2003) Variations in lung cancer risk among smokers. JNCI Journal of the National Cancer Institute 95: 470. 12644540 10.1093/jnci/95.6.470

[15] Colditz G, Atwood K, Emmons K, Monson R, Willett W, et al (2000) Harvard report on cancer prevention volume 4: Harvard Cancer Risk Index. Risk Index Working Group. Harvard Center for Cancer Prevention Cancer Causes Control 11: 477–488. 10880030 10.1023/a:1008984432272

[16] Etzel CJ, Kachroo S, Liu M, D’Amelio A, Dong Q, et al (2008) Development and validation of a lung cancer risk prediction model for African-Americans. Cancer Prev Res (Phila Pa) 1: 255–265. 10.1158/1940-6207.CAPR-08-0082 PMC2854402 19138969

[17] Spitz MR, Hong WK, Amos CI, Wu X, Schabath MB, et al (2007) A risk model for prediction of lung cancer. J Natl Cancer Inst 99: 715–726. 17470739 10.1093/jnci/djk153

[18] Tammemagi CM, Pinsky PF, Caporaso NE, Kvale PA, Hocking WG, et al (2011) Lung cancer risk prediction: Prostate, Lung, Colorectal And Ovarian Cancer Screening Trial models and validation. J Natl Cancer Inst 103: 1058–1068. 21606442 10.1093/jnci/djr173 PMC3131220

[19] Cassidy A, Myles JP, van Tongeren M, Page RD, Liloglou T, et al (2008) The LLP risk model: an individual risk prediction model for lung cancer. Br J Cancer 98: 270–276. 18087271 10.1038/sj.bjc.6604158 PMC2361453

[20] Kushi LH, Byers T, Doyle C, Bandera EV, McCullough M, et al.. (2006) American Cancer Society Guidelines on Nutrition and Physical Activity for cancer prevention: reducing the risk of cancer with healthy food choices and physical activity. CA Cancer J Clin 56: 254–281; quiz 313–254. 10.3322/canjclin.56.5.254 17005596

[21] Yun Y, Lim M, Jung K, Bae J, Park S, et al (2005) Relative and absolute risks of cigarette smoking on major histologic types of lung cancer in Korean men. Cancer Epidemiology Biomarkers & Prevention 14: 2125. 10.1158/1055-9965.EPI-05-0236 16172220

[22] World Health Organization (1994) International classification of diseases and related health problems. Geneva, Switzland.

[23] Inoue S, Zimmet P (2000) The Asian-Pacific perspective: redefining obesity and its treatment. Health Communications Australia Pty limited, Sidney, Australia.

[24] Wilson P, D’Agostino R, Levy D, Belanger A, Silbershatz H, et al (1998) Prediction of coronary heart disease using risk factor categories. Circulation 97: 1837. 9603539 10.1161/01.cir.97.18.1837

[25] Nam B (2000) Discrimination and Calibration in Survival Analysis [dissertation].

[26] Hanley JA, McNeil BJ (1982) The meaning and use of the area under a receiver operating characteristic (ROC) curve. Radiology 143: 29–36. 7063747 10.1148/radiology.143.1.7063747

[27] Colditz GA, Atwood KA, Emmons K, Monson RR, Willett WC, et al (2000) Harvard report on cancer prevention volume 4: Harvard Cancer Risk Index. Risk Index Working Group, Harvard Center for Cancer Prevention. Cancer Causes Control 11: 477–488. 10880030 10.1023/a:1008984432272

[28] Bach PB, Kattan MW, Thornquist MD, Kris MG, Tate RC, et al (2003) Variations in lung cancer risk among smokers. J Natl Cancer Inst 95: 470–478. 12644540 10.1093/jnci/95.6.470

[29] Nakaji S, Yoshioka Y, Mashiko T, Yamamoto Y, Kojima A, et al (2003) Explanations for the smoking paradox in Japan. Eur J Epidemiol 18: 381–383. 12889681 10.1023/a:1024265411218

[30] Stellman SD, Takezaki T, Wang L, Chen Y, Citron ML, et al (2001) Smoking and lung cancer risk in American and Japanese men: an international case-control study. Cancer Epidemiol Biomarkers Prev 10: 1193–1199. 11700268

[31] Takahashi I, Matsuzaka M, Umeda T, Yamai K, Nishimura M, et al (2008) Differences in the influence of tobacco smoking on lung cancer between Japan and the USA: possible explanations for the ‘smoking paradox’ in Japan. Public Health 122: 891–896. 18420238 10.1016/j.puhe.2007.10.004

[32] Marugame T, Sobue T, Satoh H, Komatsu S, Nishino Y, et al (2005) Lung cancer death rates by smoking status: comparison of the Three-Prefecture Cohort study in Japan to the Cancer Prevention Study II in the USA. Cancer science 96: 120–126. 15723657 10.1111/j.1349-7006.2005.00013.x PMC11158599

[33] Thun MJ, Hannan LM, Adams-Campbell LL, Boffetta P, Buring JE, et al (2008) Lung cancer occurrence in never-smokers: an analysis of 13 cohorts and 22 cancer registry studies. PLoS Med 5: e185. 18788891 10.1371/journal.pmed.0050185 PMC2531137

