# Supplementary material for: Individualized Risk Prediction Model for Lung Cancer in Korean Men
Source: PLoS One. 2013 Feb 7;8(2):e54823. doi: 10.1371/journal.pone.0054823 (PMC3567090; doi:10.1371/journal.pone.0054823)
Supplement: Appendix S1 — Detailed scoring system for our lung cancer risk prediction model. Table A. Application of Tables. Table B. Score sheets were developed to predict the lung cancer risk from the β-coefficient estimates in the Cox regression model (Table 3). (DOCX) [file pone.0054823.s001.docx]

**Appendix A. Application of Tables**

Step 1: Form a linear equation (A) using the β-coefficient estimates

A = 0.1668*[(Age-Mean_age_) – 0]

– 0.0020*[(Age-Mean_age_)^2^ – 107.3007]

+ 0.0*(Smoke) if Never Smoker

+ 0.4180*(Smoke – 0.1511) if Past Smoker

+ 0.4444*(Smoke – 0.0905) if Current Smoker, <0.5 pack/day

+ 0.9414*(Smoke – 0.3330) if Current Smoker, 0.5–0.99 pack/day

+ 1.3889*(Smoke – 0.1390) if Current Smoker, ≥1 pack/day

+ 0.0*(ASI) if Age at Smoking Initiation, Age ≥ 40

+ 0.2194*(ASI – 0.1441) if Age at Smoking Initiation, 30 ≤ Age < 40

+ 0.2809*(ASI – 0.3570) if Age at Smoking Initiation, 19 ≤ Age < 30

+ 0.5249*(ASI – 0.0164) if Age at Smoking Initiation, 16 ≤ Age < 19

+ 0.7120*(ASI – 0.0064) if Age at Smoking Initiation, Age < 16

+ 0.3306*(BMI – 0.0239) if BMI < 18.5

+ 0.0*(BMI) if BMI 18.5–22.9

– 0.2468*(BMI – 0.2837) if BMI 23.0–24.9

– 0.3386*(BMI – 0.2851) if BMI ≥ 25.0

+ 0.0*(PhA) if Physical Activity, none

– 0.0909*(PhA – 0.1590) if Physical Activity, light

– 0.1412*(PhA – 0.2952) if Physical Activity, moderate

– 0.0521*(PhA – 0.0676) if Physical Activity, heavy

+ 0.0*(Glucose) if Glucose < 126

+ 0.0792*(Glucose – 0.0605) if Glucose ≥ 126

Step 2: Exponential A, call it E

E = exp(A)Step 3: Calculate the probability *P* = 1 – S(t|t=8)^E^

Where S(t|t=8) is the survival probability estimate for the mean values of the risk factors in the model. Here, S(t|t=8) = 0.9983894078.

Consider a 50-year-old man, current smoker of 0.5 pack/day, age at smoking initiation of 35 years, BMI of 23 kg/m^2^, light physical activity, glucose of 120 mg/dL.

A = 0.1668*[(50 – 45) – 0] – 0.0020*[25 – 107.3007] + 0.4180*(0 – 0.1511) + 0.4444*(0 –0.0905) + 0.9414*(1 – 0.3330) + 1.3889*(0 – 0.1390) + 0.2194*(1 – 0.1441) + 0.2809*(0 – 0.3570) + 0.5249*(0 – 0.0164) + 0.7120*(0 – 0.0064) + 0.3306*(0 – 0.0239) – 0.2468*(1 – 0.2837) – 0.3386*(0 – 0.2851) – 0.0909*(1 – 0.1590) – 0.1412*(0 – 0.2952) – 0.0521*(0 – 0.0676) + 0.0792*(0 – 0.0605) = 1.28023

E = exp(A) = exp(1.28023) = 3.59748

P = 1- S(t|t=8)^E^ = 1 – 0.9983894078 ^(3.59748)^ = 0.005781964, for 0.5782%

chance of developing lung cancer over 8 years.

**Appendix B.**

Score sheets were developed to predict the lung cancer risk from the β-coefficient estimates in the Cox regression model (Table 3)

Table S1. Lung cancer risk for each risk factor in points

| Risk factor | Points* |
| --- | --- |
| Age (years) |  |
| 30–34 | -30 |
| 35–39 | -19 |
| 40–44 | -9 |
| 45–49 | 0 |
| 50–54 | 8 |
| 55–59 | 15 |
| 60–64 | 21 |
| 65–69 | 25 |
| 70–74 | 29 |
| 75–80 | 32 |
| Smoke |  |
| Never | 0 |
| Past | 4 |
| Current, <0.5 pack/day | 4 |
| Current, 0.5–0.99 pack/day | 9 |
| Current, ≥1 pack/day | 14 |
| Age at smoking initiation (current smokers only) |  |
| Age ≥ 40 | 0 |
| 30 ≤ Age < 40 | 2 |
| 19 ≤ Age < 30 | 3 |
| 16 ≤ Age < 19 | 5 |
| Age < 16 | 7 |
| BMI, kg/m^2^ |  |
| <18.5 | 3 |
| 18.5–22.9 | 0 |
| 23.0–24.9 | -2 |
| ≥25.0 | -3 |
| Physical activity |  |
| No | 0 |
| Light | -1 |
| Moderate | -1 |
| Heavy | -1 |
| Fasting Glucose levels, mg/dL |  |
| <126 | 0 |
| ≥126 | 1 |

* Standardized points are proportional to the *β* coefficients in the risk prediction model.
